# Host Specificity of *Epiplema albida*: A Potential Biological Control Agent for Sri Lankan Privet in the Mascarene Islands

**DOI:** 10.3390/insects8030077

**Published:** 2017-07-28

**Authors:** Richard H. Shaw, Matthew J. W. Cock

**Affiliations:** CABI, Bakeham Lane, Egham, Surrey TW20 9TY, UK; m.cock@cabi.org

**Keywords:** *Ligustrum robustum* subsp. *walkeri*, Lepidoptera, Uraniidae, Epipleminae, Oleaceae, La Réunion

## Abstract

*Epiplema albida* (Hampson) (Lepidoptera: Uraniidae, Epipleminae) from Sri Lanka, was studied to assess its safety for use as a biological control agent for Sri Lankan privet, *Ligustrum robustum* subsp. *walkeri* (Oleaceae) in La Réunion and other Mascarene Islands. Larval no-choice feeding tests using newly hatched larvae, larval development tests, and multiple choice oviposition tests were used. Adult females of *E. albida* are shown to have highly selective oviposition behaviour and the species is physiologically restricted to very few hosts for feeding and development. The risk to key test plants in La Réunion is minimal, so this species can be considered for use as a biological control agent there, but would need further evaluation for potential use elsewhere.

## 1. Introduction

Invasive alien species have been described as one of the biggest threats to the flora and fauna of islands [1,2]. Introduced Asian *Ligustrum* spp. (Oleaceae) are invasive in other parts of the world [3]: *L. sinense* Lour., has been widely introduced and is reported invasive in Argentina, Puerto Rico, mainland USA, Australia, New Zealand and the Pacific; *L. lucidum* W.T. Aiton has also been widely introduced and is considered invasive in South Africa, mainland USA, Argentina and Australia; and *L. obtusifolium* Siebold and Zucc. is reported invasive in parts of mainland USA. The European *L. vulgare* L. is reported invasive in South Africa, and parts of North and South America, Australia and New Zealand [3]. Biological control of *L. sinense* is being evaluated in the USA ([4] and subsequent papers). Sri Lankan privet (or Ceylon privet), *Ligustrum robustum* subsp. *walkeri* (Decne.) P.S. Green, is a woody shrub or small tree indigenous to Sri Lanka, and a serious invasive alien plant on the Mascarene Islands [3,5], which because of their high levels of endemicity have international conservation significance as biodiversity hotspots [6].

The problem is particularly acute on the island of La Réunion, a French Overseas Department, which has the largest area of intact vegetation of the Mascarene Archipelago [7]. Sri Lankan privet is thought to have arrived in La Réunion in the 1960s, but it was not until the 1980s that the real threat to the fragile ecosystem of this island was realized [8].

In La Réunion, only intensive management on a massive scale would offer any chance of controlling the plant and allowing native species to regain and retain their place in the ecosystem. The wide distribution and difficulty of access contribute to make traditional weed control techniques, including felling and herbicide treatment, almost impossible, and the plant continues to spread almost unhindered although the pace of spread seems to have slowed compared with that seen in the 1990s [9]. Biological control has the advantage of being largely unhindered with respect to access and, unlike herbicides, targets individual species without having any impact on valued indigenous species. CABI undertook to investigate the scope for classical biological control of Sri Lankan privet on behalf of La Région Réunion. Field surveys of the natural enemies of *L. robustum* in its indigenous range were made and have been reported for the hill country of Sri Lanka [10].

Sri Lankan privet, *Ligustrum robustum* subsp. *walkeri*, was originally described as a valid species, but has also been treated as a variety of *L. robustum* (Roxb.) Blume, and is currently accepted as a subspecies [11,12,13]. The indigenous distribution of *L. robustum* is restricted to India, Sri Lanka, and Bangladesh to Vietnam. Within this range, subspecies *robustum* was described from Bangladesh (Silhet) and is found from northeast India to Thailand and Vietnam [12]. Subspecies *walkeri* is restricted to Sri Lanka [11,12,14,15]. The southern Indian population was treated as a subspecies of *L. robustum*, but is now considered a separate species, *L. perrottetii* A. DC. [14,15].

As part of the natural enemy surveys that were carried out, dried leaf samples were collected for molecular analysis. The material introduced and established in the Mascarene Islands was shown to be derived from the Sri Lankan population of *L. robustum* subsp. *walkeri*, that it had undergone little or no loss of genetic diversity since introduction, and it had not introgressed with germplasm from the other two alien *Ligustrum* species present on La Réunion [16].

The fact that the target plant is a minor element of the flora in its indigenous range [10], suggested that the prospects for biological control of *L. robustum* were good. An effective flower-feeder or seed-feeder would make a good biological control agent since preventing long range dispersal would allow land managers to concentrate on the existing restricted infestations. This could also be achieved through targeting of the seedling stage of the plant. However, a suite of agents damaging different parts and stages of Sri Lankan privet is likely to be more effective than one agent in controlling this woody plant.

As a result of our surveys in Sri Lanka, a defoliating moth, *Epiplema albida* (Hampson) (Lepidoptera: Uraniidae, Epipleminae), was selected for evaluation as a potential biological control agent for introduction to La Réunion. The genus *Epiplema* includes *E. fulvilinea* Walker, whose larvae are a recognized pest of *Gmelina arborea* Roxb. ex Sm. (Lamiaceae) seedlings in Kerala, India [17] and *E. dohertyi* Warren, a minor pest of coffee (Rubiaceae) in Kenya [18], i.e., some species are capable of causing significant damage to their host plants. At least two indigenous parasitoids were recorded to commonly attack larvae in Sri Lanka, but parasitism of eggs and pupae was not investigated [19]. This suggests that parasitoids may have an important role in keeping the population of *E. albida* in check in Sri Lanka, and that freed from these, *E. albida* could build up high population densities if introduced into La Réunion. However, other members of the Epipleminae are present in the Mascarenes [20] and an investigation of likely predators and parasites in the area of release would be appropriate. Such natural enemies can limit the efficacy of a biological control agent after release and are the cause of approximately 20% of recorded biocontrol agent failures [21,22].

Cock and Shaw [19] report on the biology of *E. albida*: females lay an average of 80 ova on leaves, there are four instars of leaf-feeding larvae, pupation is in or on the soil, and the whole life cycle takes just 34 days on average. Thus under favourable conditions, this species has the potential to increase in numbers very rapidly if unchecked. More than 10 generations could be completed in a year at 20 °C based on our controlled conditions, and possibly more in La Réunion where the normal temperature range is 21–26 °C. Here we report our studies on the host specificity of *E. albida*, as a basis for assessing its safety for introduction as a biological control agent in La Réunion.

## 2. Materials and Methods 

Early stages of *E. albida* were field collected from multiple locations in Sri Lanka [10] taken to the UK and held in the CABI UK quarantine facility, in a controlled environment room at 20 °C (±2.2 °C) with a 12 hours daylight: 12 hours darkness regime and 60–80% humidity. A culturing method was developed [19]. Culturing and all studies were carried out under these conditions in quarantine over a period of 21 months.

A test plant list of 88 species was compiled based on phylogeny [23] and availability (Table 1, 21 species of Oleaceae, Table 2, 23 species of other families of Lamiales, and Appendix A 44 species of other orders, mainly economic species). The classification of the order Lamiales has been in a state of flux and so test plants were selected to cover the main groups of Oleaceae and representatives of other families also placed in the Lamiales when the test plant list was developed. Additional species from other orders, in particular a selection of economic species were also tested to satisfy the requirements of the authorities in La Réunion. Many members of the tribe Oleeae are known to have similar iridoid glucosides [24], so these are the genera most likely to be included in the host range of a specialist *Ligustrum*-feeder. The key test plant species for this project were the native Mascarene Oleaceae: *Noronhia broomeana* Horne ex Oliv. (a La Réunion-endemic with only 11 individuals known in the wild), *N. emarginata* (Lam.) Thouars (a Madagascan native naturalized in Mauritius and La Réunion), *Olea europaea* subsp. *africana* (Mill.) P.S. Green and *O. lancea* Lam. (Mascarene endemics, although subsp. *africana* may no longer be recognised, and *O. lancea* may now be a synonym of the widespread *O. capensis* L.; Tropicos [25] and The Plant List [13] offer conflicting views). Significant damage to any of these would not be acceptable, although damage to other *Ligustrum* spp. would be.

Test plants were grown in 11 cm pots in John Innes No. 2 potting compost and watered when needed. Non-chemical pest management was applied if required but if pest issues became serious then the plants were not used for experiments. The selection of plant species to be used in each set of tests on a given date was influenced by their availability and priority. Studies included larval feeding no-choice and choice tests, larval development and oviposition choice tests.

### 2.1. Larval No-Choice Feeding Tests

A small bouquet of cut test plant foliage with stems wrapped in moist cotton wool held with cling-film was presented to individual newly-hatched larva (<1 day old) in a 9 cm Petri dish. Care was taken to ensure that a similar amount of plant material was included in each bouquet irrespective of the different morphology of the test plants, equivalent to two Sri Lankan privet leaves. Feeding levels were recorded on a scale of 0–5 where 0 = no feeding, t = trace feeding, 1 = 1–5% consumed, 2 = 6–10%, 3 = 11–20%, 4 = 21–50% and 5 ≥ 50%. Each test was replicated at least six times and if any equivocal results were recorded then additional replicates were added (Table 1). Each batch of tests was run alongside equivalent controls of Sri Lankan privet, and continued until all larvae died or completed development. The number of larvae alive was recorded at least every two days and a record of the maximum feeding score was made (to overcome issues with varying material replacement times and lack of synchrony of feeding score recording which would have made mean scoring inappropriate). The whole study was carried out over a 20 month period. Results were tabulated for comparison but not analyzed further.

### 2.2. Larval Development Tests on Excised Foliage

Following on from the no choice studies detailed above, plant material was provided until the surviving larvae pupated. The number of pupae formed and adults that emerged after feeding on each plant species was recorded. All test foliage was obtained from potted plants with the exception of *L. ovalifolium,* for which leaves were collected from plants growing nearby and brought into the laboratory. Results were tabulated for comparison but not analyzed further.

### 2.3. Larval Development Tests on Potted Plants

In order to check that using cut plant material did not affect the feeding activity of the larvae, more realistic tests were carried out using the limited supply of potted key test plants: *O. lancea*, *O. europaea* subsp. *africana* and *N. broomeana*, with Sri Lankan privet as controls (Table 1). Newly-hatched first instar larvae were transferred in batches of 20 to healthy potted test plants with young foliage, which were held in a ventilated cylinder cage 30 cm in diameter and 45 cm tall. The number alive and the level of feeding damage were recorded at 4–5 day intervals, and the results plotted graphically.

### 2.4. Larval Choice-Tests

Individual larvae were presented with a choice between excised Sri Lankan privet foliage and similar material from each of those plant species on which some feeding had been recorded in no-choice tests, apart from *N. emarginata*, for which insufficient test plants were available. These were set up in the same way as for the larval development tests on excised foliage described above. The number of larvae alive and their feeding score was recorded daily. The significance of any difference in feeding levels on the target versus the test plant was determined with paired t-tests.

### 2.5. Single-Choice Oviposition Tests

Since the adult oviposition site is the primary determinant of the host plant of progeny, experiments were carried out to determine whether mated *E. albida* females would show selective oviposition behaviour in 50 cm × 40 cm × 50 cm ventilated perspex cages.

Four male and two female newly-emerged moths were released into a cage. They were presented with a choice between a potted Sri Lankan privet plant and a test plant of about 30 cm height (shorter plants were raised so that they were presented at 30 cm height). In the case of *L. ovalifolium,* the plant material was excised since the plants were too large to fit in a cage and this previously had been found to be acceptable to the adult moths. Honey feeders were also provided for the adults to feed and refreshed as necessary over the five days of the test. The plants were then removed and the egg number and location (upper or lower leaf) recorded.

### 2.6. Multiple-Choice Oviposition Tests

Multiple-choice oviposition tests were carried out to provide a more realistic situation than individual choice tests. The methods were as in the single-choice oviposition tests above, using the same cages and numbers of moths, but with each cage containing plants of *L. robustum, O. lancea*, *O. europea* subsp. *africana, L. ovalifolium, N. broomeana* and *N. emarginata* arranged randomly at equivalent heights. There were 12 non-simultaneous replicates. Student’s t-test was used to test the significance of the difference in egg counts between the controls and the test plant that received the most eggs.

## 3. Results

### 3.1. Larval No-Choice Feeding Tests

In total, 864 larvae were subjected to no-choice tests on 89 species of test plants (Table 1 and Table 2, Appendix A). Only eight test plant species had a feeding score above 1 (>5% consumed) and these are presented in Table 3. All other test plants has feeding scores of less than 1, and all test larvae on these plants died by day 5. All of the plants which were fed upon are members of the subtribe Ligustrinae, apart from *Olea lancea* and *Noronhia* spp. which are in the subtribe Oleinae.

### 3.2. Larval Development Tests on Excised Foliage

Of those test plant species which were accepted in no-choice tests, only seven species including the target were suitable for some larvae to complete development to adults (Table 3). Over 50% of the larvae completed development on the control Sri Lankan privet, three *Syringa* spp. and *N. emarginata.*

### 3.3. Larval Development Tests on Potted Plants

**Figure 1:**
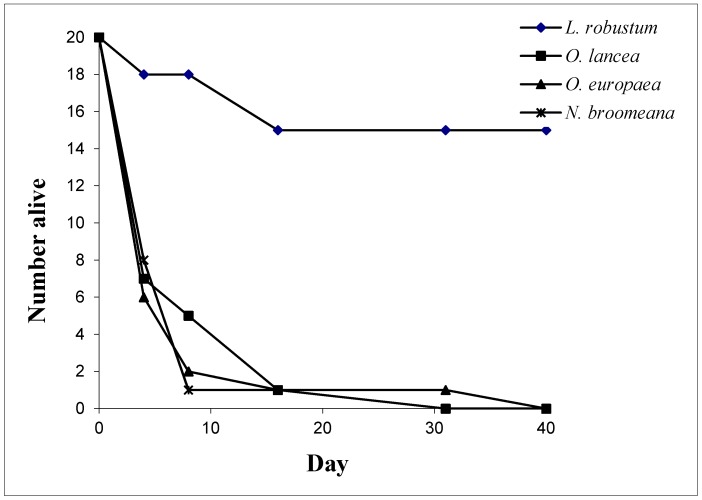
Survivorship curves for 1st instar *Epiplema albida* larvae feeding on potted key test plants: *Olea lancea*, *O. europaea* subsp. *africana* and *Noronhia broomeana*, with *Ligustrum robustum* subsp. *walkeri* as controls.

Figure 1 shows that, although capable of surviving longer on some test plants than observed in the cut foliage studies, the larvae of *E. albida* were incapable of completing development on the La Réunion Oleaceae tested. One moth did manage to reach pupation on *O. europea* subsp. *africana*, but the pupa failed to emerge.

### 3.4. Larval Choice-Tests

The mean feeding scores for day 4 are presented in Table 4. Four days represents a point at which the larvae have fed substantially, but before the food supply had begun to deteriorate. Damage scores on the control (*L. robustum*) and *L. ovalifolium* were not significantly different, but the damage scores on the other test plants were significantly lower than on *L. robustum.*

### 3.5. Single-Choice Oviposition Tests

These results are summarized in Table 5. The overall mean number of eggs laid on the target privet species was 75.6 (SE ± 12.8), with only two out of 20 plants receiving no eggs. Excluding the test using both species of privet, 1512 eggs were laid on *L. robustum* in the presence of a non-target plant without a single egg being laid on the test plants other than *Ligustrum* spp. Based on these preliminary results, we switched to multiple-choice oviposition tests.

### 3.6. Multiple-Choice Oviposition Tests

A total of 1191 eggs were laid in the 12 replicates of the multiple choice oviposition tests. Over 96% were laid on the target Sri Lanka privet, 3.1% on *L. ovalifolium*, seven on *O. lancea*, and none on the other test plants (Table 6). The difference between the mean number of eggs laid on Sri Lanka privet and on *L. ovalifolium* was highly significant (t = 4.69; df = 11, *p* = 0.0006). When eggs were laid on non-target plants, they were always found in areas where previous larval feeding had taken place and never on the surface of a healthy leaf. This appeared to be because the feeding ‘craters’ made in the relatively more fleshy leaves of these non-target plants provided an attractive oviposition site, resembling the crenulations of *L. robustum* leaves, but no systematic observations were made on this point. Most of the non-target oviposition was on *L. ovalifolium*, but a comparatively very small number of eggs was recorded from two replicates of *O. lancea*.

## 4. Discussion

*Epiplema albida* has been shown to be highly selective in its egg laying behaviour and physiologically restricted to very few hosts for feeding and development. To be considered safe for introduction into La Réunion, feeding damage on non-target *Ligustrum* spp. would be considered acceptable, but significant damage on indigenous Oleaceae (*Noronhia* spp., *Olea* spp.) would not be.

The Oleaceae test plant list (Table 1) provide the basis for a critical evaluation of the potential host range, while test plants in Table 2 and Appendix A are less critical. The results of the no-choice feeding tests and subsequent development studies (Table 3) indicate that larvae of *E. albida* have a highly restricted host plant range and are only capable of developing to adult on a small subset of the subtribe Ligustrinae plus *N. emarginata* of the Oleinae. The successful rearing of the moth on leaves of *N. emerginata* is of note since *N. emarginata* is a Madagascan endemic naturalized in La Réunion, but the La Réunion endemic *N. broomeana* was not accepted. These results are supported by the larval development test on potted plants, where development on the control was excellent, but most larvae on three critical test plants (*O. lancea*, *O. europaea* subsp. *africana* and *N. broomeana*) were dead within ten days (Figure 1). The relatively low successful development rate on *L. ovalifolium* may be linked to the use of field collected leaves in winter, which probably would have been less suitable for larval development. Larval choice tests demonstrated a strong and significant preference for the target species, except in the case of the European *L. ovalifolium* which was moderately acceptable. Unfortunately, due to lack of tests plants, *N. emarginata* was not included in the larval choice tests.

Larvae will not normally move from the plant on which oviposition takes place, and the results of the oviposition tests show that *E. albida* is highly selective as to which plants it chooses to lay eggs on. The overwhelming preference for oviposition in multiple choice tests was for the control, but a few eggs were laid on *L. ovalifolium* and seven eggs on *O. lancea*, but none on the other test plants including *N. emarginata* (Table 5). In a single choice situation two *O. lancea* plants did not receive any eggs compared to an average of 57 on the two controls (Table 5), suggesting that the eggs laid in the multiple choice arena were accidental. Thus, of all the test plants, only *L. ovalifolium* was acceptable for both oviposition and larval development, and none of the key test plants or any of the other plants tested were suitable oviposition plants for *E. albida* in our studies. Similarly, Paynter et al. [27] developed an index of the risk of non-target attack, based on the multiplicative combination of acceptance in host-specificity tests (i.e., oviposition, neonate acceptance and larval development in our study). They found a transition between values of 0.21 and 0.33 for the index, below which there was no non-target attack in the field, and above which there was almost certain to be non-target attack. In our results, the critical test plants (indigenous *Olea* spp. and *Noronhia* spp.) all scored zero, supporting our conclusions stated above. The score for *L. ovalifolium* was well below the threshold, so non-target effects would not be expected. Although the scores for *Syringa* spp. were well above the threshold, these did not include any oviposition tests, as damage to this genus is not considered of importance in La Réunion. Were *E. albida* to be considered for use in other countries, further tests on any Ligustrinae of local importance would be needed [28].

In a preliminary study, defoliation by *E. albida* was shown to be capable of killing potted plants [29] and significant impacts have been recorded on other species of potted privets [30,31]. However, studies by Obeso and Grubb [32] on *L. sinense* showed no effects of defoliation on fruit maturation. Whether impacts on seedlings would be adequate to limit the spread of the plant is unknown. Equally, whether a defoliator can have significant impact on *L. robustum* is difficult to predict. We could not test efficacy against mature plants in the quarantine laboratory due to availability and space limitations. Nonetheless there are many defoliators of trees that have significant detrimental impact on their hosts, especially in outbreak conditions, so there is no reason to believe *E. albida* won’t have the desired effect were it to be released.

## 5. Conclusions

This study was completed without any follow-up implementation in La Réunion. It remains to be seen whether *E. albida* will be used for the biological control of Sri Lankan privet on La Réunion or the other Mascarene Islands where it remains a highly invasive element of the natural habitat, displacing indigenous biodiversity. The data presented here evaluate the risks to non-target plants that introduction of *E. albida* would present in the Mascarenes, particularly La Réunion, demonstrate that it would be safe to introduce, and has the potential to have useful impact on Sri Lankan privet. *Epiplema albida* is potentially of use for the biological control of other invasive *Ligustrum* spp. in other parts of the world, but this would require further study on the suitability of the target species, and host-specificity would need to be reassessed in relation to local concerns, particularly indigenous Oleaceae.

## Figures and Tables

**Table 1:** Oleaceae test plants for *Epiplema albida* indicating which species were used in what numbers in each test: (1) larval no-choice feeding test, (2) larval development tests on excised foliage, (3) larval development tests on potted plants, (4) larval choice-tests, (5) single-choice oviposition tests, and (6) multiple-choice oviposition tests. Nomenclature has been checked against Tropicos [25] and The Plant List [13], and classification is based on Wallander [26].

		Host Specificity Tests					
Tribe Subtribe	Species (variety)	1	2	3	4	5	6
Oleeae							
Ligustrinae	*Ligustrum robustum* subsp. *walkeri*	153	24	20	– ^c^	– ^c^	12
	*L. ovalifolium*	18	18		12	2	12
	*L. japonicum*	6					
	*L. lucidum*	6					
	*Syringa* × *josiflexa* (Bellicent)	12	12				
	*S. pubescens* subsp. *microphylla* (Superba)	12					
	*Syringa wolfii*	6	6				
	*S. reticulata* subsp. *pekinensis*	6	6			2	
	*S. meyeri* (Palibin) ^b^	13	13				
	*S. vulgaris* (Madame Lemoine)	18			6		
Fraxininae	*Fraxinus excelsior*	12					
Oleinae	*Olea europaea* subsp. *africana* ^a^	53		24	10		12
	*O. europaea* subsp. *europaea*	5			10	7	12
	*O. lancea*^a^	56	56	21	10	2	12
	*Osmanthus heterophyllus*	9			6		
	*O. delavayi*	6			6		
	*Noronhia broomeana* ^a^	23	6	12	6	7	12
	*N. emarginata* (from La Réunion) ^a^	16	16				12
Jasmineae	*Jasminum nudiflorum*	6					
	*J. polyanthum*	6					
	*J. humile*	6					
Forsythieae	*Forsythia* sp. (mini gold)	18					

^a^ Key test plant species; ^b^ This may be a synonym of *S. pubescens* [13,25]; ^c^ Controls.

**Table 2:** *Epiplema albida* test plants of the Lamiales other than Oleaceae (a further 44 species from 27 families of other orders, mainly plants of economic importance were included and are listed in Appendix A).

Order	Family	Species (Variety)
Lamiales	Acanthaceae	*Acanthus mollis*, *Hypoestes sanguinolenta*
	Bignoniaceae	*Dolichandra unguis-cati*, *Tecoma capensis*
	Gesneriaceae	*Streptocarpus rexii*
	Lamiaceae	*Clerodendrum bungei*, *C. costatum* (=*C. cunninghamii*), *C. thomsoniae*, *C. tomentosum*
	Orobanchaceae	*Orobanche* sp.
	Pedaliaceae	*Sesamum indicum*
	Phrymaceae	*Mimulus guttatus*
	Plantaginaceae	*Antirrhinum majus*, *Hebe ligustrifolia*, *H. vernicosa*, *Hebe* sp. (lilac colour), *Hebe* sp. (Wiri splash), *Plantago major, Veronica chamaedrys*
	Scrophulariaceae	*Buddleja davidii*, *Scrophularia nodosa*
	Stilbaceae	*Nuxia verticillata*
	Verbenaceae	*Lantana camara*

**Table 3:** Feeding scores, survival and development of first instar *Epiplema albida* larvae in no-choice feeding tests and subsequent larval development tests.

Test Plant	Number of Larvae Tested	Max. Feeding Score	No. Alive on Day 5	Number Pupated	Number of Adults	% that Completed Development
*Ligustrum robustum* subsp. *walkeri*	24	5	20	17	13	54
*Ligustrum ovalifolium*	18	5	3	3	1	6
*Syringa* × *josiflexa*	12	5	8	8	5	42
*Syringa wolfii*	6	4	1	1	1	17
*Syringa meyeri*	13	5	10	10	10	77
*Syringa pekinensis*	6	5	4	3	3	50
*Olea lancea*	56	2	1	0	0	0
*Noronhia broomeana* ^a^	6	2	0	0	0	0
*N. emarginata*	16	4	14	10	9	56

^a^ The leaves used were particularly young.

**Table 4:** Damage recorded on day 4 in choice tests carried out on *Epiplema albida* larvae in Petri dishes (for the analysis trace damage was recorded as 0.5 and samples with dead larvae were not included).

Test Plant Species	Replicates	Mean Damage Score Control (±SE)	Mean Damage Score Test sp. (±SE)	Paired *t*-Test Significance (Degrees of Freedom)
*Ligustrum ovalifolium*	12	1.75 ± 1.22	1.13 ± 1.05	*p* > 0.05 (11)
*Olea lancea*	10	1.95 ± 1.01	0.60 ± 0.84	*p* < 0.05 (9)
*O. europaea* subsp. *europaea*	10	2.80 ± 1.03	0.10 ± 0.21	*p* < 0.001 (9)
*O. europaea* subsp. *africana*	10	2.10 ± 0.57	0.20 ± 0.26	*p* < 0.001 (9)
*Syringa vulgaris*	6	2.42 ± 1.02	0.0 ± 0.2	*p* < 0.01 (5)
*Noronhia broomeana*	6	3.50 ± 0.84	0.58 ± 0.38	*p* < 0.001 (5)
*Jasminum polyanthus*	6	3.00 ± 0	0.08 ± 0.2	*p* < 0.001 (5)
*Osmanthus heterophyllus*	6	3.17 ± 0.75	0.00 ± 0	*p* < 0.001 (5)
*O. delavayi*	6	3.33 ± 0.82	0.00 ± 0	*p* < 0.001 (5)

**Table 5:** The mean number of eggs per plant laid by adult *Epiplema albida* during preliminary single–choice oviposition (control *Ligustrum robustum* subsp. *walkeri*).

Test Plant Species	Replicates	Mean No. Eggs on Control (±SE)	Mean No. Eggs on Test Plant (±SE)
*Ligustrum ovalifolium*	2	188 ± 60.1	17 ± 4
*Olea lancea*	2	57 ± 8.5	0
*O. europaea* subsp. *europaea*	7	44 ± 31	0
*Syringa pekinensis*	2	83 ± 19.1	0
*Noronhia broomeana*	7	78 ± 56.3	0

**Table 6:** The number of eggs laid by *Epiplema albida* on test plants in 12 multiple choice oviposition tests. In addition to these results, there was no oviposition in any of the 12 trials on *Olea europaea* subsp. *europaea*, *O. europaea* subsp. *africana*, *Noronhia broomeana* or *N*. *emarginata*.

Test Plant	Total Number of Eggs Laid	Average Number of Eggs	Minimum Number of Eggs	Maximum Number of Eggs	Standard Error
*Ligustrum robustum* subsp. *walkeri*	1147	176.5	20	221	68
*Ligustrum ovalifolium*	37	5.4	0 (×8)	21	6.3
*Olea lancea*	7	0.5	0 (×10)	6	0.9

## Appendix A

List of further plants of economic importance from 25 families included in host range testing (see Table 2).

Anacardiaceae: *Mangifera indica*

Annonaceae: *Annona squamosa*

Apiaceae: *Daucus carota*

Apocynaceae: *Asclepias curassavica*, *Camptocarpus mauritianus*, *Carissa macrocarpa* (=*C. grandiflora*), *C. spinarum* (=*C. xylopicron*), *Ochrosia borbonica*

Araceae: *Anthurium andreanum*

Araliaceae: *Fatsia japonica*

Arecaceae: *Chamaedorea elegans*

Brassicaceae: *Nasturtium officinale*

Bromeliaceae: *Ananas comosus*

Caricaceae: *Carica papaya*

Cucurbitaceae: *Cucurbita pepo*

Fabaceae: *Acacia giraffae*, *A. heterophylla*, *Phaseolus vulgaris*, *Tamarindus indica*

Geraniaceae: *Pelargonium* spp.

Malvaceae: *Dombeya burgessiae*

Moraceae: *Ficus* sp.

Musaceae: *Musa acuminata*

Myrtaceae: *Psidium guajava*

Orchidaceae; *Vanilla planifolia*

Passifloraceae: *Passiflora aurantia*, *P. amethystina*, *P. edulis*

Poaceae *Hordeum vulgare*, *Saccharum officinarum*, *Zea mays*

Rosaceae: *Prunus padus*, *P. persica*

Rutaceae: *Citrus limon*, *Citrus* sp., *Fortunella* sp.

Sapindaceae: *Dimocarpus longan*, *Litchi sinensis*

Convolvulaceae: *Ipomea purpuria*

Rubiaceae: *Coffea canephora*, *Coffea* sp.

Solanaceae: *Nicotiana tabacum*, *Solanum melongena*

Vitaceae: *Vitis vinifera*
